# Antimicrobial susceptibility of *Neisseria gonorrhoeae* in Barcelona during a five-year period, 2013 to 2017

**DOI:** 10.2807/1560-7917.ES.2020.25.42.1900576

**Published:** 2020-10-22

**Authors:** Paula Salmerón, Belén Viñado, Rachid El Ouazzani, Marta Hernández, María Jesús Barbera, Mireia Alberny, Mireia Jané, Nieves Larrosa, Tomás Pumarola, Yannick Hoyos-Mallecot, Judit Serra-Pladevall

**Affiliations:** 1 Microbiology Department, Vall d’Hebron Hospital Universitari, Barcelona, Spain; 2 Drassanes-Vall d'Hebron Sexually Transmitted Infections Unit, Department of Infectious Diseases, Vall d’Hebron Hospital Universitari, Barcelona, Spain; 3 Universitat de Barcelona, Barcelona, Spain; 4 Medical Management of Primary Care Services, Institut Català de la Salut (ICS), Barcelona, Spain; 5 Agència de Salut Pública de Catalunya, Generalitat de Catalunya, Barcelona, Spain; 6 Microbiology group, Vall d’Hebron Institut de Recerca (VHIR), Vall d’Hebron Hospital Universitari, Barcelona, Spain; 7 Universitat Autònoma de Barcelona (UAB), Bellaterra, Spain

**Keywords:** *Neisseria gonorrhoeae*, antimicrobial resistance, extended-spectrum cephalosporins, azithromycin

## Abstract

**Introduction:**

Increasing rates of antimicrobial resistance in *Neisseria gonorrhoeae* cause problems for treating gonorrhoea.

**Aim:**

This observational study aimed to describe isolates from all patients found infected with *N. gonorrhoeae*, in Barcelona, Spain, between 2013 and 2017, and with available antimicrobial susceptibility data.

**Methods:**

Minimum inhibitory concentrations (MICs) of penicillin (PEN), cefixime (CFM), ceftriaxone (CRO), azithromycin (AZM), ciprofloxacin (CIP), spectinomycin (SPT), fosfomycin (FOF) and gentamicin (GEN) were determined by E-test. Susceptibility was assessed using clinical breakpoints from the European Committee on Antimicrobial Susceptibility Testing. Time trends for PEN, CFM, AZM and CIP were investigated using logistic regression.

**Results:**

Of 1,979 patients with infection (2,036 isolates), 1,888 (95.4%) were men. Patient median age was 32 years. The proportions of isolates resistant to extended-spectrum cephalosporins were low, with 0.3% (5/1,982) resistant to CRO and 4.9% (98/1,985) to CFM. AZM resistance prevalence was 2.7% (52/1,981), including 16 isolates detected in 2016 and 2017, with high-level resistance. For CIP, 51.3% (1,018/1,986) of isolates were resistant, and for PEN, 20.1% (399/1,985). All isolates were susceptible to SPT. MIC_50_ and MIC_90_ values of GEN were 4 and 6 mg/L and of FOF 12 and 24 mg/L, respectively. Between 2013 and 2017, PEN and CFM resistance rates each decreased from 28.1% (92/327) to 12.2% (70/572) and from 8.3% (27/327) to 4.4% (25/572) (p ≤ 0.0073). In contrast, AZM resistance prevalence appeared to increase from 1.5% in 2014 (5/340) to 3.0% (17/572) in 2017. No trend was identified for CIP.

**Conclusion:**

Antimicrobial susceptibility surveillance is important to timely detect new phenotypes and trends.

## Introduction

Gonorrhoea is the second most reported bacterial sexually transmitted infection (STI), after chlamydia. Untreated gonorrhoea can lead to pelvic inflammatory disease and infertility in women and epididymitis and orchitis in men. In the European Union/European Economic Area countries, a total of 89,239 confirmed cases of gonorrhoea were reported in 2017 with an overall rate of 22.2 cases per 100,000 population [1]. This represented a 17% increase over the previous year, which was particularly striking in certain groups, such as men who have sex with men (MSM), who represented almost half of the cases in 2017, and the 25–34-year-old population [1].

In Spain, 8,722 cases of gonococcal infection were reported in 2017 (rate: 18.74 per 100,000 inhabitants), with a very wide range of infection incidence among the different regions of the country, from 2.44 and 48.50 cases per 100,000 inhabitants. The highest rates were registered in Catalonia (48.50), Balearic Islands (41.79) and Madrid (28.48) [2]. Of the 3,622 cases reported in Catalonia in 2017, men accounted for 82% of the diagnoses. Data from 1,136 patients (31%) could be collected: 44% were MSM followed by heterosexual men (22%) and women (20%) [3].

*Neisseria gonorrhoeae*, the bacterial species responsible for gonorrhoea, has developed resistance to the different families of antibiotics used in the past, challenging future treatment. Currently, extended-spectrum cephalosporins (ESC) are the last-line treatment option for *N. gonorrhoeae* infection. Unfortunately, strains showing resistance to ESC have been reported worldwide [4-6]. In 2017, the European Centre for Disease Control and Prevention (ECDC) reported a resistance rate to cefixime of 1.9% within the European Union/European Economic Area countries and no isolates showing resistance to ceftriaxone [7]. The resistance rate to azithromycin was 7.5%; and high-level resistance (minimum inhibitory concentration (MIC) ≥ 256 mg/L) was detected in seven isolates [7].

This pathogen has moreover been able to acquire or develop nearly all known mechanisms of antimicrobial resistance: target modification, inactivation of the antimicrobial by enzymatic means, decreased influx of antimicrobials, and increased efflux of antimicrobials [8]. This situation highlights the importance of carrying out resistance monitoring programmes, in order to update therapeutic guidelines and to timely detect the emergence of multidrug-resistant strains.

Nowadays, most of the treatment guidelines recommend dual antimicrobial therapy (500 mg intramuscular ceftriaxone + 1 g or 2 g oral azithromycin) [9-12]. The rationale for gonococcal combination therapy using different antimicrobials with different mechanisms of action is to potentially mitigate the spread of antimicrobial resistance [13]. Combination therapy has been reported as possibly related to the decline in prevalence of *N. gonorrhoeae* isolates with decreased susceptibility to ceftriaxone (DSC), defined as a MIC > 0.032 mg/L [14,15].

The aim of this study is to describe antimicrobial susceptibility of *N. gonorrhoeae* in isolates collected between 2013 and 2017 in Barcelona, Spain.

## Methods

### Study population

The study population consisted of all patients who were diagnosed with a *N. gonorrhoeae* infection and with isolates for which antimicrobial susceptibility data were available. When patients tested positive for gonorrhoea on multiple sites and more than one culture was obtained, only the genital culture was included in the study.

Patients were attended in three different clinical settings: Drassanes-Vall d'Hebron Sexually Transmitted Infections unit, different medical departments of Vall d'Hebron University Hospital and primary healthcare units in Barcelona, Spain. Together these settings cover 1,200,000 of the 1,620,343 inhabitants of Barcelona (74.1%).

### Culture

Urethral, rectal, vaginal, endocervical, and/or pharyngeal samples were cultured on selective Thayer–Martin medium and incubated at 35–37 °C in a 5% CO_2_ atmosphere for 24–48 hours. Probable *N. gonorrhoeae* strains were identified by oxidase reaction (oxidase-positive) and mass spectrometry (MALDI-TOF, Vitek MS system, Biomérieux, Marcy-I´Étoile, France). *N. gonorrhoeae* strains were subcultured in order to obtain fresh colonies for antimicrobial susceptibility testing (AST) and frozen at −80 °C in trypticase soy broth with 20% glycerol.

### Antimicrobial susceptibility

AST was performed on fresh colonies (<24 hours). The MICs of penicillin, ceftriaxone, cefixime, ciprofloxacin, azithromycin, spectinomycin, gentamicin, and fosfomycin were determined by means of the E-test method (bioMérieux, France), as described by the Clinical and Laboratory Standard Institute (CLSI) [16]. Interpretation was performed using clinical breakpoints from the European Committee on Antimicrobial Susceptibility Testing (EUCAST) [17], except for gentamicin and fosfomycin, since cut-off points for these antibiotics are not established by EUCAST (nor by CLSI). *N. gonorrhoeae* ATCC 49226 was used as a reference strain for antimicrobial susceptibility testing.

### Descriptive and statistical analysis

Descriptive analyses of the study population were performed. Statistical analyses were conducted using Stata (StataCorp, College Station, Texas, US). Trends of penicillin, cefixime, azithromycin and ciprofloxacin resistance over the study period were calculated using logistic regression analyses.

Determinants for resistance for penicillin (MIC > 1 mg/L), cefixime (MIC > 0.125 mg/L), azithromycin (epidemiological cut-off (ECOFF) > 1 mg/L) and ciprofloxacin (MIC > 0.06 mg/L) were identified using logistic regression analyses. Univariable and multivariable analyses were performed. Differences with p < 0.05 were considered statistically significant. As there were very few strains that reached the 0.125 mg/L threshold of ceftriaxone resistance, we did not determine associations between potential factors for ceftriaxone resistance and resistance to this antibiotic.

### Ethical statement

As this was a retrospective study, no ethical approval was needed. All data related to patients were coded to maintain confidentiality.

## Results

Between 2013 and 2017, 2,054 strains were isolated from 1,979 patients. Eighteen strains were excluded because, in 16 patients, *N. gonorrhoeae* was recovered from two different body sites, and in one patient, gonococcus was recovered from three different sites. In the end, susceptibility testing was performed on 2,036 *N. gonorrhoeae* strains isolated from 1,979 patients, because 47 patients presented two different episodes and five patients presented three episodes during the study period.

Of the 1,979 patients included, 1,888 (95.4%) were from men. 1,292 (65.3%) were tended in the Drassanes-Vall d'Hebron Sexually Transmitted Infections Unit. Furthermore, 573 patients (29.0%) presented in primary healthcare units and 114 (5.8%) in other medical departments of Vall d'Hebron University Hospital. For 1,555 patients with data on age available, the median age was 32 years, and 1,173 (75.4%) were between 20 and 40 years old. A total of 1,598 (78.5%) of the 2,036 isolates studied were from urethral samples and 1,888 (95.4%) patients were men (Table 1).

**Table 1:** Characteristics of patients included in the study on antimicrobial susceptibility of *Neisseria gonorrhoeae* isolates, Barcelona, Spain, 2013−2017 (n = 1,979 patients)

Characteristics	2013		2014^a^		2015^b^		2016		2017		TOTAL^c^	
	Number	%	Number	%	Number	%	Number	%	Number	%	Number	%
Numbers of patients and isolates^d^												
Isolates	329	100	340	100	339	100	447	100	581	100	2,036	100
Patients	321	100	326	100	328	100	423	100	581	100	1,979	100
Sex												
Men	303	94.4	306	93.9	320	97.6	400	94.6	559	96.2	1,888	95.4
Women	18	5.6	18	5.5	8	2.4	23	5.4	22	3.8	89	4.5
Unknown	0	0.0	2	0.6	0	0.0	0	0.0	0	0.0	2	0.1
Age in years												
< 20	NA	NA	14	6.1	9	2.8	12	2.8	9	1.5	44	2.8
20–29	NA	NA	73	31.7	118	36.8	153	36.2	236	40.6	580	37.3
30–39	NA	NA	91	39.6	121	37.7	167	39.5	214	36.8	593	38.1
40–49	NA	NA	39	17.0	52	16.2	68	16.1	84	14.5	243	15.6
≥ 50	NA	NA	13	5.7	21	6.5	23	5.4	38	6.5	95	6.1
Median (range)	NA	NA	32 (14–74)	NA	32 (14–72)	NA	32 (4–75)	NA	31 (5–75)	NA	32 (4–75)	NA
Clinical setting												
Drassanes STI unit	214	66.7	232	71.2	209	63.7	275	65.0	362	62.3	1,292	65.3
Primary healthcare units	90	28.0	76	23.3	100	30.5	118	27.9	189	32.5	573	29
Other HUVH departments	17	5.3	18	5.5	19	5.8	30	7.1	30	5.2	114	5.8
Specimen												
Urethral/balanoprepucial	261	79.3	245	72.1	274	80.8	365	81.7	453	78.0	1,598	78.5
Rectal	38	11.6	53	15.6	48	14.2	56	12.5	88	15.1	283	13.9
Endocervical/vaginal	16	4.9	11	3.2	10	2.9	21	4.7	11	1.9	69	3.4
Pharynx	10	3.0	11	3.2	7	2.1	5	1.1	29	5.0	62	3.0
Other^e^	4	1.2	20	5.9	0	0.0	1	0.2	0	0.0	24	1.2

HUVH: Vall d'Hebron University Hospital; NA: not available; STI: sexually transmitted infection.

^a^ Age data available from 230 patients (from April 2014 to December 2014).

^b^ Age data available from 321 patients.

^c^ Age data available from 1,555 patients.

^d^ Number of isolates can be greater than number of patients because over the study period several patients had more than one episode of gonorrhoea.

^e^ Other includes: synovial fluid, abdominal abscess, perianal exudates.

The denominators for the percentages concerning patients’ characteristics are the total numbers of patients with information available on the characteristic in question, for the year or period specified in the column header. Denominators for specimen percentages are total number of isolates.

Antimicrobial susceptibility data during the study period are shown in Table 2.

**Table 2:** Antimicrobial susceptibility of *Neisseria gonorrhoeae* isolates collected in Barcelona, Spain, 2013−2017 (n = 2,036)

Antimicrobial	MIC_50_ in mg/L	MIC_90_ in mg/L	MIC range in mg/L	Susceptibility category^a^					
				Susceptible		Intermediate		Resistant	
				Number	%	Number	%	Number	%
PEN^b^	0.25	12	0.002 – > 32	135	6.8	1,451	73.1	399	20.1
CRO^c^	0.016	0.047	< 0.016 – 0.38	1,977	99.7	0	0.0	5	0.3
CFM^b^	0.016	0.094	< 0.016 – 0.38	1,887	95.1	0	0.0	98	4.9
AZM^d^	0.125	0.25	< 0.016 – > 256	1,929	97.4	0	0.0	52	2.6
CIP^e^	0.38	> 32	< 0.002 – > 32	965	48.6	3	0.2	1,018	51.3
SPT	8	12	1.5 – 64	2,036	100	0	0.0	0	0.0
GEN^f^	4	6	0.25– 24	NA	NA	NA	NA	NA	NA
FOF^g^	12	24	0.064 – 128	NA	NA	NA	NA	NA	NA

AZM: azithromycin; CIP: ciprofloxacin; CFM: cefixime; CRO: ceftriaxone; ECOFF: epidemiological cut-off value; EUCAST: European Committee on Antimicrobial Susceptibility Testing; FOF: fosfomycin; GEN: gentamicin; MIC: minimum inhibitory concentration; NA: not applicable; PEN: penicillin; SPT: spectinomycin.

^a^ According to EUCAST (2019) clinical breakpoints.

^b^ Antimicrobial susceptibility testing for PEN and CFM was performed on 1,985 isolates.

^c^ Antimicrobial susceptibility testing for CRO was performed on 1,982 isolates.

^d^ Antimicrobial susceptibility testing for AZM was performed on 1,981 isolates.

^e^ Antimicrobial susceptibility testing for CIP was performed on 1,986 isolates.

^f^ GEN was determined in the period from 2013 to 2016.

^g^ FOF was determined in 2015 and 2016.

The distribution of MIC values by year for ceftriaxone, cefixime and azithromycin is shown in the Figure (panels A, B and C respectively). Most of the isolates (69.6%; 1,380/1,982) showed a ceftriaxone MIC ≤ 0.016 mg/L. The percentage of ceftriaxone resistant isolates (defined as MIC > 0.125 mg/L) appeared to decrease from 0.6% (2/324) in 2013 and 2014 to 0.2% (1/408) in 2016 (p = 0.4637). No isolates resistant to ceftriaxone were found either in 2015 or 2017. The average prevalence of decreased susceptibility to ceftriaxone (DSC; 0.032 < MIC < 0.125 mg/L) throughout the study period was 15.8% (314/1,982), seemingly declining from 2013 to 2015 (from 24.1% (78/324) to 7.4% (25/339)) but in 2016 and 2017 the proportions of isolates with DSC returned to higher levels, 19.1% (78/408) and 14.3% (82/572), respectively.

**Figure:**
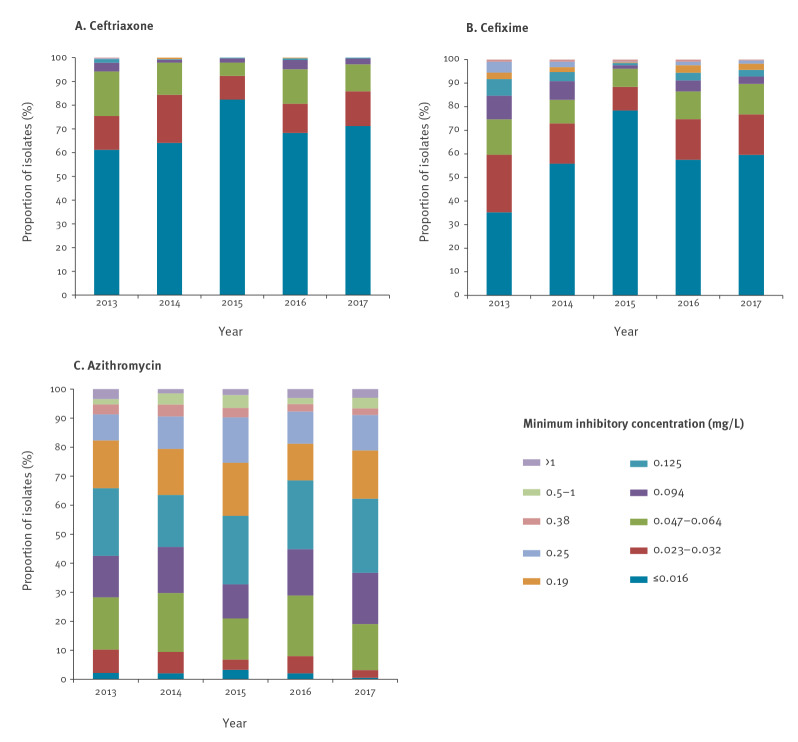
Proportion of *Neisseria gonorrhoeae* isolates with different minimum inhibitory concentrations (mg/L) for (A) ceftriaxone (n = 1,982 isolates), (B) cefixime (n = 1,985 isolates) and (C) azithromycin (n = 1,981 isolates), by year, Barcelona, Spain, 2013−2017

EUCAST: European Committee on Antimicrobial Susceptibility Testing; MIC: minimum inhibitory concentration.

According to EUCAST, the MIC breakpoints for resistance to ceftriaxone and cefixime are identical and are MIC > 0.125 mg/L. The epidemiological cut-off for azithromycin is > 1mg/L.

The average percentage of isolates with cefixime resistance (MIC > 0.125 mg/L) at 4.9% was higher than the average percentage with ceftriaxone resistance at 0.3% (Table 2). Despite this, cefixime resistance rate decreased from 8.3% (27/327) in 2013 to 4.4% (25/572) in 2017 (p = 0.0073). For both ESC, the proportion of isolates with MIC ≤ 0.016 mg/L appeared to increase from 2013 to 2015, from 35.2% (115/327) to 78.5% (266/339) for cefixime and from 61.1% (198/324) to 82.3% (279/339) for ceftriaxone (Figure).

For azithromycin, most of isolates (54.6%; 1,082/1,981) showed MIC between 0.094 and 0.19 mg/L. Annual MIC_50_ and MIC_90_ values remain the same during the study period, 0.125 and 0.25 mg/L, respectively. Nevertheless, the proportion of resistant isolates, according to the EUCAST breakpoint (ECOFF > 1mg/L) seemed to increase slightly from 1.5% (5/340) in 2014 to 3.0% (17/572) in 2017 (p = 0.5295). A total of 16 strains showed high-level azithromycin-resistance (MIC ≥ 256mg/L): seven in 2016 and nine in 2017. These 16 strains accounted for almost a third (16/52) of all azithromycin resistant strains. No high-level azithromycin-resistant gonococcal isolates were found from 2013 to 2015.

However, the proportion of isolates showing both DSC and azithromycin resistance appeared to decrease from 1.5% (5/329) in 2013 to 0.3% (2/581) in 2017.

The prevalence of resistance to ciprofloxacin and penicillin was 51.3% and 20.1%, respectively (Table 2). For penicillin, the resistance rate presented a statistically significant decline over the study years (p = 0.0000), from 28.1% (92/327) in 2013 to 12.2% (70/572) in 2017. In addition, 14.7% (300/2,036) of isolates were penicillase-producing *N. gonorrhoeae* (PPNG). For ciprofloxacin, the resistance rate fluctuated between 48.8 and 56.8% during the study period, with no clear trend. All isolates were susceptible to spectinomycin (Table 2). The MIC_50_ and MIC_90_ values for gentamicin were 4 and 6 mg/L, while for fosfomycin these were 12 and 24 mg/L, respectively (Table 2).

Table 3 summarises the determinants of resistance to the different antibiotics tested during the study.

**Table 3:** Determinants, according to logistic regression analysis, of resistance to penicillin (MIC > 1 mg/L), cefixime (MIC > 0.125 mg/L), azithromycin (ECOFF > 1 mg/L) and ciprofloxacin (MIC > 0.06 mg/L) in *Neisseria gonorrhoeae* isolates from primary healthcare units and Drassanes-Vall d’Hebron sexually transmitted infection unit, Barcelona, Spain, 2013–2017 (n = 2,036)

Variable	Number of resistant isolates	Total isolates tested	%	Univariable		Multivariable		Number of resistant isolates	Total isolates tested	%	Univariable		Multivariable	
				OR (95%CI)	p value	OR (95%CI)	p value				OR (95%CI)	p value	OR (95%CI)	p value
**Antibiotic**	**Penicillin**							**Cefimixime**						
Year														
2013	92	327	28.1	1 (ref)	0.0000	1 (ref)	0.0000	27	327	8.3	1 (ref)	0.0064	1 (ref)	0.0073
2014	88	339	26.0	0.93 (0.66–1.33)		0.97 (0.68–1.38)		18	339	5.3	0.63 (0.33–1.20)		0.67 (0.35–1.29)	
2015	73	339	21.5	0.73 (0.51–1.05)		0.70 (0.49–1.01)		5	339	1.5	0.19 (0.07–0.50)		0.20 (0.07–0.52)	
2016	76	408	18.6	0.61 (0.42–0.86)		0.59 (0.42–0.85)		23	408	5.6	0.60 (0.32–1.12)		0.61 (0.32–1.16)	
2017	70	572	12.2	0.34 (0.24–0.49)		0.33 (0.23–0.47)		25	572	4.4	0.43 (0.23–0.80)		0.43 (0.23–0.80)	
Total	399	1,985	NA	NA	NA	NA	NA	98	1,985	NA	NA	NA	NA	NA
Sex														
Male	388	1,895	20.5	1 (ref)	0.1786	1 (ref)	0.1067	88	1,896	4.6	1 (ref)	0.0074	1 (ref)	0.0303
Female	11	88	12.5	0.55 (0.23–1.31)		0.49 (0.20–1.17)		10	87	11.5	3.36 (1.38–8.16)		2.72 (1.10–6.72)	
Total	399	1,983^a^	NA	NA	NA	NA	NA	98	1,983^a^	NA	NA	NA	NA	NA
Clinical setting														
DVHSTI (n = 1,340^b^)	242	1,303	18.6	1 (ref)	0.0041	1 (ref)	0.0006	46	1,303	3.5	1 (ref)	0.0064	1 (ref)	0.0050
PH (n = 579^b^)	138	566	24.4	1.41 (1.12–1.79)		1.53 (1.20–1.95)		36	565	6.4	1.86 (1.19–2.92)		1.91 (1.22–3.01)	
Total	380^c^	1,869^c^	NA	NA	NA	NA	NA	82^c^	1,868^c^	NA	NA	NA	NA	NA
**Antibiotic**	**Azithromycin**							**Ciprofloxacin**						
Year														
2013	11	322	3.4	1 (ref)	0.5295	1 (ref)	0.5939	162	325	49.8	1 (ref)	0.0771	1 (ref)	0.0846
2014	5	340	1.5	0.42 (0.15–1.23)		0.45 (0.15–1.30)		167	340	49.1	1.01 (0.74–1.39)		1.06 (0.77–1.45)	
2015	7	339	2.1	0.60 (0.23–1.57)		0.60 (0.23–1.57)		192	338	56.8	1.45 (1.06–1.99)		1.44 (1.05–1.98)	
2016	12	408	2.9	0.81 (0.34–1.89)		0.81 (0.35–1.90)		213	407	52.3	1.11 (0.82–1.50)		1.11 (0.82–1.50)	
2017	17	572	3	0.87 (0.40–1.88)		0.85 (0.39–1.84)		284	576	49.3	1.002 (0.758–1.325)		0.98 (0.74–1.30)	
Total	52	1,981	NA	NA	NA	NA	NA	1,018	1,986	51.3	NA	NA	NA	NA
Sex														
Male	50	1,891	2.6	1 (ref)	0.5410	1 (ref)	0.6184	965	1,896	50.9	1 (ref)	0.9312	1 (ref)	0.9832
Female	2	88	2.2	1.57 (0.37–6.65)		1.45 (0.34–6.22)		53	88	60.2	0.98 (0.55–1.73)		0.99 (0.56–1.77)	
Total	52	1,979^a^	NA	NA		NA	NA	1,018	1,984^a^	NA	NA	NA	NA	NA
Clinical setting														
DVHSTI (n = 1,340^b^)	28	1,302	2.2	1 (ref)	0.0207	1	0.0272	612	1,303	47.0	1	0.0000	1 (ref)	0.0000
PH (n = 579^b^)	23	563	4.1	1.94 (1.11–3.39)		1.89 (1.07–3.31)		332	567	58.6	1.63 (1.34–1.99)		1.63 (1.33–1.99)	
Total	51^c^	1,865^c^	NA	NA	NA	NA	NA	944^c^	1,870^c^	NA	NA	NA	NA	NA

CI: confidence interval; DVHSTI: Drassanes-Vall d’Hebron sexually transmitted infection unit; ECOFF: epidemiological cut-off value; MIC: minimum inhibitory concentration; NA: not applicable; OR: odds ratio; PH: primary healthcare unit.

^a^ There are two patients for whom sex is not known.

^b^ Number of isolates received by the setting.

^c^ Isolates from Vall d’Hebron University Hospital are not included in the statistical analyses relating to clinical setting.

Antimicrobial resistance rates were compared between isolates from primary healthcare units and the Drassanes-Vall d’Hebron STI unit. Resistance rates of all antibiotics were higher in primary healthcare units than in Drassanes-Vall d‘Hebron STI unit, being statistically significant for penicillin (odds ratio (OR): 1.53; 95% confidence interval (CI): 1.20–1.95; p = 0.0006), cefixime (OR: 1.91; 95%CI: 1.22–3.01; p = 0.0050), azithromycin (OR: 1.89; 95%CI: 1.07–3.31; p = 0.0272) and ciprofloxacin (OR: 1.63; 95%CI: 1.33–1.99; p = 0.0000) (Table 3).

## Discussion

This study describes antimicrobial surveillance data from 2,036 *N. gonorrhoeae* isolates in Barcelona during a 5-year period. To our knowledge, this is the first report in Spain describing antimicrobial resistance of such a considerable number of *N. gonorrhoeae* isolates. Other studies have been published previously [18,19], but the amount of strains included was much lower. Our findings show that the rate of ceftriaxone resistance remains low and stable. These results agree with data from the ECDC [7]. From 2013 to 2015 the number of isolates with MIC ≤ 0.016 mg/L of ceftriaxone and cefixime seemed to increase progressively. However, in 2016 and 2017 we observed an important decrease in the percentage of strains with MIC ≤ 0.016 mg/L and an increase in strains with higher MICs, which concur with a report that isolates with DSC have been emerging in Europe in recent years [15].

In our work, we were able to observe a decrease in the resistance rate of both ESC throughout the study. The reason that this decrease is statistically significant only for cefixime may be due to the small number of ceftriaxone resistant strains.

Since dual therapy (ceftriaxone 500 mg + azithromycin 1 g) was implemented in Barcelona in 2012, resistance to ceftriaxone has started to decrease: 2.8% in 2012 (data not shown) to 0.0% in 2017. This could be explained by the change in treatment regimen whereby, until 2011, gonorrhoea was treated with ceftriaxone 250 mg. On the other hand, the resistance rate of azithromycin has increased slightly from 1.5% in 2014 to 3.0% in 2017 and high-level resistant isolates (MIC ≥ 256 mg/L) were first detected in 2016 and 2017. An increase in azithromycin rate has been documented in several European countries [15].

Although the European and Spanish guidelines still recommend dual therapy [12,20], the increase in azithromycin resistance detected worldwide, not only in *N. gonorrhoeae* but also in other STIs such as *Mycoplasma genitalium* [21], brings into question the advisability of this therapeutic strategy. In fact, since 2019, the British Association for Sexual Health and HIV (BASHH) has recommended monotherapy with ceftriaxone 1 g [22]. However, some studies support that the selection/induction of azithromycin resistance of *N. gonorrhoeae* by the use of the current dual therapy is limited [23], and it may be associated with the general use of azithromycin for respiratory infections or the treatment of non-gonococcal urethritis [13].

The overall azithromycin resistance rate in our study was 2.6% based on the EUCAST breakpoint (ECOFF is 1 mg/L). This percentage is lower than that reported by other Spanish groups such as Cobo et al. [18], who showed a resistance percentage of 13.8% in Almería. On the other hand, Fuertes de Vega et al. reported a resistance rate of 5.2% in Barcelona [19], which is more similar to our results. We must bear in mind that the cut-off point used by these authors is that of prior EUCAST report versions (0.5 mg/L). If we analyse the azithromycin resistance rate of our study according to this cut-off, the percentage of resistance strains would be 4.1%.

The difference in the percentage of azithromycin resistance between different regions of Spain could be due to divergence in treatment patterns or the circulation of different genotypes with greater ability to develop antimicrobial resistance.

On other hand, the occurrence of extensively drug-resistant (XDR) strains of *N. gonorrhoeae* reported in countries such as the United Kingdom and Australia in 2018 is concerning [24]. These strains show a high level of azithromycin resistance and they are also resistant to ceftriaxone, resulting in resistance to the first line dual therapy for gonorrhoea (ceftriaxone intramuscularly and azithromycin orally) recommended by European, Australian and World Health Organization guidelines. The appearance and dissemination of these type of strains compromises the successful treatment of this infection. This highlights the need to keep the capability to culture *N. gonorrhoeae*, in order to monitor antibiotic susceptibility and rapidly detect the emergence of XDR strains.

Although the resistance rate to ciprofloxacin observed in this study is similar to that of neighbouring countries [25], it is higher than that reported in eastern European countries such as Ukraine [26].

The breakpoints of gentamicin are not yet established by EUCAST. In this study it appears that *N. gonorrhoeae* does not have high MIC values for gentamicin (MIC_50_ = 4mg/L and MIC_90_ = 6mg/L). These values are similar to those reported previously and suggest that gentamicin can be a low-cost and efficacious alternative therapy [27]. In addition, other studies support the in vitro activity of gentamycin, used in combination with cefixime or ertapenem, to control the spread of multidrug-resistant (MDR) and XDR *N. gonorrhoeae* strains [28].

Similar to gentamicin, no clinical breakpoints for fosfomycin exist. The review conducted by Tesh et al. suggests that fosfomycin can be an alternative treatment for gonococcal infection [29]. In this review, isolates with a MIC < 16 mg/L were considered as susceptible, while those with MICs between 32 and 64 mg/L were moderately susceptible. These MICs are similar to those found by our study, but it is necessary to establish susceptibility breakpoints in order to understand how to appropriately dose fosfomycin to treat *N. gonorrhoeae* infections [29]. 

It is surprising that the resistance rates of almost all antimicrobials tested were higher in isolates from primary healthcare than in those from the STI unit. One reason that might explain this is that 70% of the Drassanes-Vall d'Hebron STI Unit patients are MSM. Although we do not have access to this information, we hypothesise by deduction that the majority of patients who attend primary healthcare units are men who have sex with women (MSW) and if so, this might suggest that there are different *N. gonorrhoeae* populations circulating in the two populations.

One limitation of this study is that antimicrobial susceptibility tests of *N. gonorrhoeae* were performed mainly in symptomatic patients, since they are the ones from whom a sample is collected for culture. Asymptomatic patients are diagnosed by nucleic acid amplification tests (NAAT). Another limitation is that when *N. gonorrhoeae* was isolated in multiple sites in one patient, antimicrobial susceptibility was only performed on genital samples. This could lead to the loss of strains with higher MIC values in pharyngeal carriers and consequently to underestimate antimicrobial resistance. Moreover, due to the retrospective nature of the study, there is a lack of demographic and epidemiological information.

Although our results are only representative of Barcelona, the patients included in the current study represent 62.1% of those reported in Catalonia [30].

In conclusion, we analysed the susceptibility of 2,036 isolates in Barcelona from 2013 to 2017. We were able to observe that susceptibility to ceftriaxone remains high and resistance has decreased since dual therapy was implemented in 2012. However, azithromycin resistance increased during the study period and high-level azithromycin resistant strains were isolated for the first time in 2016.

Our study highlights the need to monitor antibiotic susceptibility and to perform molecular typing studies at a national level, which would allow identifying temporal and geographical changes, to detect the emergence and dissemination of new strains, and to maintain therapeutic guidelines updated.
